# Parallel Adaptive Divergence among Geographically Diverse Human Populations

**DOI:** 10.1371/journal.pgen.1002127

**Published:** 2011-06-16

**Authors:** Jacob A. Tennessen, Joshua M. Akey

**Affiliations:** Department of Genome Sciences, University of Washington, Seattle, Washington, United States of America; Georgia Institute of Technology, United States of America

## Abstract

Few genetic differences between human populations conform to the classic model of positive selection, in which a newly arisen mutation rapidly approaches fixation in one lineage, suggesting that adaptation more commonly occurs via moderate changes in standing variation at many loci. Detecting and characterizing this type of complex selection requires integrating individually ambiguous signatures across genomically and geographically extensive data. Here, we develop a novel approach to test the hypothesis that selection has favored modest divergence at particular loci multiple times in independent human populations. We find an excess of SNPs showing non-neutral parallel divergence, enriched for genic and nonsynonymous polymorphisms in genes encompassing diverse and often disease related functions. Repeated parallel evolution in the same direction suggests common selective pressures in disparate habitats. We test our method with extensive coalescent simulations and show that it is robust to a wide range of demographic events. Our results demonstrate phylogenetically orthogonal patterns of local adaptation caused by subtle shifts at many widespread polymorphisms that likely underlie substantial phenotypic diversity.

## Author Summary

Identifying regions of the human genome that differ among populations because of natural selection is both essential for understanding evolutionary history and a powerful method for finding functionally important variants that contribute to phenotypic diversity and disease. Adaptive events on timescales corresponding to the human diaspora may often manifest as relatively small changes in allele frequencies at numerous loci that are difficult to distinguish from stochastic changes due to genetic drift, rather than the more dramatic selective sweeps described by classic models of natural selection. In order to test whether a substantial proportion of interpopulation genetic differences are indeed adaptive, we identify loci that have undergone moderate allele frequency changes in multiple independent human lineages, and we test whether these parallel divergence events are more frequent than expected by chance. We report a significant excess of polymorphisms showing parallel divergence, especially within genes, a pattern that is best explained by geographically varying natural selection. Our results indicate that local adaptation in humans has occurred by subtle, repeated changes at particular genes that are likely to be associated with important morphological and physiological differences among human populations.

## Introduction

Although the predominant population genetics model of adaptation assumes novel advantageous alleles sweep to fixation [1], [2], most putative examples of adaptive divergence between human populations lack the full signature of a classic hard sweep [2]–[10]. In fact, classic sweeps may have played a negligible role in the evolutionary changes that have occurred since the most recent common human ancestor [10], prompting the question of whether the moderately large allele frequency differences observed among modern human populations at a small proportion of loci are indeed adaptive. These divergent loci may indicate more complex and subtle modes of selection, but it is often difficult to demonstrate with statistical confidence that they are not merely the tail end of a stochastic neutral distribution. However, if selection acts independently on the same loci in different geographical locations, data from multiple populations can be leveraged to provide strong evidence for non-neutral evolution. Such parallel adaptation among populations of the same species has been identified in sticklebacks [11], [12], whitefish [13], Drosophila [14]–[16], and other taxa [17], [18]. The extent to which the same genes underlie repeated adaptive events is unclear, but a growing number of observations suggest that parallel evolution at the molecular level may be quite prevalent [19]–[21], and from first principles it seems especially likely when selection acts on the same standing genetic variation in closely related populations [2], [22]. Demonstration of parallel evolution among populations provides strong support for the hypothesis that repeated selection of the same alleles in distinct environments is an important mechanism of local adaptation. Studying this evolutionary process can also generate a list of candidate sites that likely have functional phenotypic consequences and provide insight into which environments present similar selective pressures.

**Figure 1:**
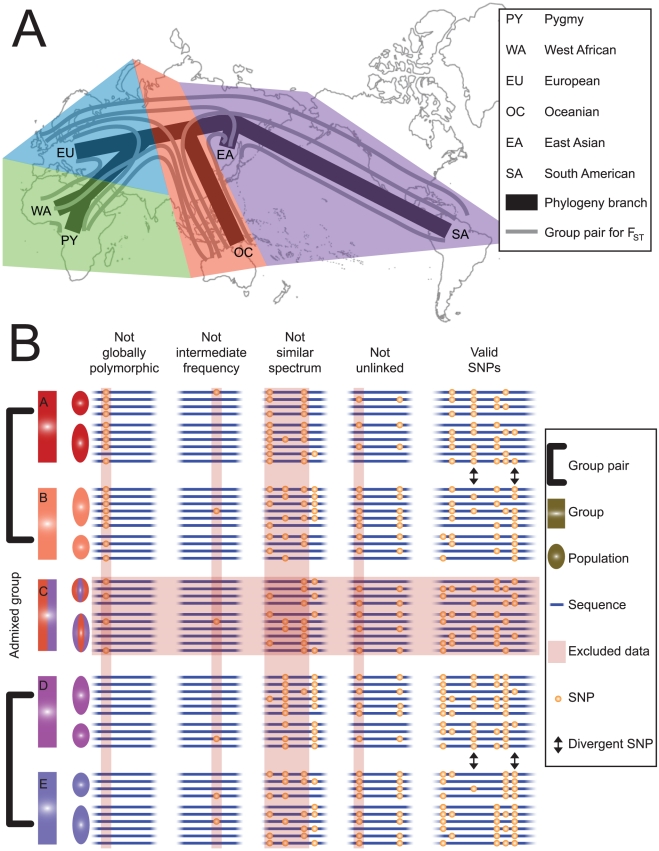
Schematic diagram of our methodology. (A) Unrooted phylogenetic relationship among the six demographic groups examined in this study. A phylogenetically independent divergence comparison can be made between any two sets of pairwise F_ST_ values corresponding to two group pairs that do not appear together in the same colored region; thus, fifteen divergence comparisons are possible. (B) Hypothetical dataset comprising a single divergence comparison showing parallel divergence. Five groups (A–E) are shown, formed of two populations each; only the four groups that fall into two clear, phylogenetically independent group pairs are retained, while admixed group C is excluded. SNPs are excluded for four reasons. First, if SNPs are invariant in an entire group pair (“not globally polymorphic”), because these may represent recent mutations instead of ancestral polymorphisms. Second, if SNPs show a global minor allele frequency less than 0.4 (“not intermediate frequency”), because these may have had skewed ancestral frequencies that were less likely to become divergent via drift. Third, if SNPs cause the site frequency spectra of some groups to differ substantially from those of other groups (“not similar spectrum”), because these may reflect differences in ascertainment or demographic history. Fourth, if SNPs are in linkage disequilibrium with an included SNP (“not unlinked”), because these are not independent. Eight valid SNPs remain. Two divergent SNPs at a threshold of 25% (a higher threshold than we used in practice) in both group pairs are indicated. Both are parallel divergent SNPs showing the same orientation (A/D, B/E), so the orientation skew is 100%.

In this study, we develop a novel approach to test the hypothesis that parallel adaptive evolution has shaped extant patterns of human genomic variation. Our method evaluates a set of independent SNPs genotyped in populations that can be clustered into at least four groups forming two or more phylogenetically distinct, allopatric group pairs (Figure 1). The goal is to test whether the same SNPs show high divergence in phylogenetically independent contrasts [23] between pairs of groups more often than expected under neutrality. To this end, we calculate pairwise F_ST_ for all SNPs between all pairs of groups. For each pair of groups, we identify divergent SNPs that exceed the 95^th^ percentile of F_ST_ values, and define parallel divergent SNPs as those that are divergent in two independent group pairs. A significant excess of parallel divergent SNPs is interpreted as evidence for parallel adaptive divergence. We demonstrate substantial parallel adaptation in human populations and we characterize the genomic and geographical patterns of the parallel divergent SNPs.

## Results

### Dataset Suitability

We applied our method to the Human Genome Diversity Project (HGDP) data, which consists of approximately 1,000 individuals genotyped for over 650,000 SNPs [24]. We only analyzed a subset of these data that met our assumptions of independence, chosen as follows. We combined 19 of the HGDP populations, consisting of 343 individuals, into six ecologically and genetically distinct groups (Figure 1A). Fifteen divergence comparisons are possible in this dataset, where each divergence comparison contrasts F_ST_ values calculated in two phylogenetically independent pairs of groups. Although it is difficult to completely rule out cryptic gene flow, we carefully examined these groups for any evidence of admixture. Previous population structure analysis indicates that the particular samples that constitute these groups are distinct lineages [25]. Possible gene flow between Pygmies and West Africans is irrelevant to this analysis as these sister populations are never assigned to different groups in a divergence comparison. The only other detectable gene flow signature among these populations is low-level introgression from East Asia into Oceania [25]; therefore the three divergence comparisons that could potentially be confound by this introgression, all comparing East Asia–South America F_ST_ against F_ST_ between Oceania and another population, are evaluated with this caveat in mind. To supplement these prior population structure results, we confirmed that the established phylogenetic topology has the significantly highest likelihood (Shimodaira and Hasegawa tests; p<0.001). Furthermore, after removing divergent SNPs exceeding the 95^th^ percentile, we regressed the ranks of F_ST_ values at remaining SNPs for each group pair against the corresponding ranks for other group pairs and found that non-divergent F_ST_ values are uncorrelated (Bonferroni-corrected p>0.05 for all divergence comparisons). This result suggests that admixture has had a negligible effect on these populations, since high levels of gene flow would cause SNPs to show relatively similar F_ST_ values in multiple group pairs.

To address potential confounding factors in our analysis, we only considered a subset of all HGDP SNPs to meet the assumptions of our neutral model (Figure 1B). One confounding factor is variation among SNP frequencies in the common human ancestor population, since the expected divergence due to genetic drift depends on the ancestral minor allele frequency; therefore SNPs with skewed global frequency, a proxy for the ancestral frequency, were excluded. Another confounding factor is mutation rate; therefore only globally polymorphic SNPs showing variation within all group pairs were used, to maximize the probability that alleles are identical by descent and not new mutations. We identified 111,724 HGDP SNPs that were intermediate frequency and globally polymorphic. Within this set, linked SNPs are not independent and ascertainment bias may be more pronounced in some populations [26]; therefore only SNPs showing low linkage disequilibrium with each other and collectively similar site frequency spectra in all groups were analyzed together (Figure S1). We randomly selected 1000 subsets of SNPs (mean = 26,864 SNPs; range = 25,371 to 27,770 SNPs) meeting these criteria. Thus, the theoretically expected number of parallel divergent SNPs in a divergence comparison is 67.16 ( = 26,864*0.05^2^; Text S1). An ideal dataset for this analysis would employ full sequence data rather than SNPs and potentially larger sample sizes than the 28 to 116 individuals per population that we used. In the absence of such a dataset, it is unlikely that any sampling scheme will produce a truly independent set of SNPs or precise estimates of allele frequencies, but our method represents a way to eliminate the most egregious potential errors while still retaining a larger number of SNPs for analysis.

### Parallel Divergence among Human Populations

**Figure 2:**
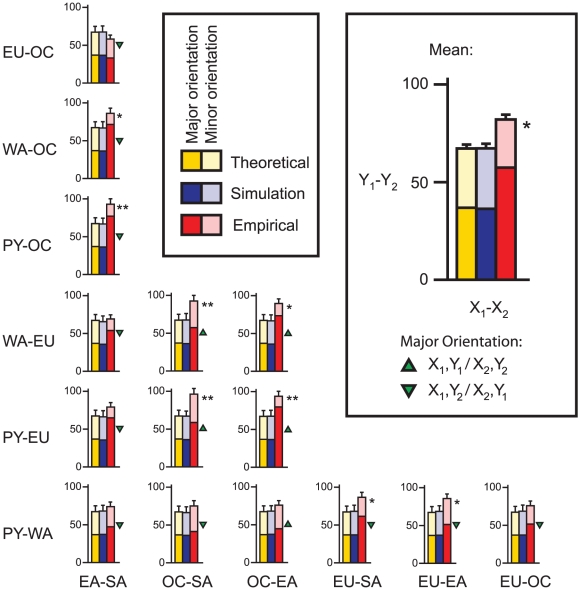
Mean number of parallel divergent SNPs for all fifteen divergence comparisons at a 5% threshold. Groups are abbreviated as in Figure 1A. Theoretical expectations (the threshold squared times the total) are shown in yellow (error bars = theoretical standard deviation; Text S1). Mean results from 1000 simulated samples under the neutral *standard* model are shown in blue (error bars  =  standard deviation). Observed empirical counts are shown in red (error bars  =  standard deviation among 1000 samples of independent SNPs). For all but one divergence comparison, more parallel divergent SNPs than expected were observed, indicative of parallel adaptation. For each bar, the shading indicates the orientation skew; the darker shade represents the number of SNPs showing the major orientation, and the lighter shade represents the number of SNPs showing the minor orientation. A skew deviating from 50% indicates that each group in the y-axis group pair tended to have relatively similar allele frequencies as a particular group in the x-axis group pair, as indicated by the green triangles. A single asterisk (*) indicates a significant excess of parallel divergent SNPs relative to the *standard* simulations (≥81 SNPs; p<0.05), while a double asterisk (**) indicates significance both relative to the *standard* simulations and in Bonferroni-corrected Fisher's exact tests (≥91 SNPs; p<0.05).

**Figure 3:**
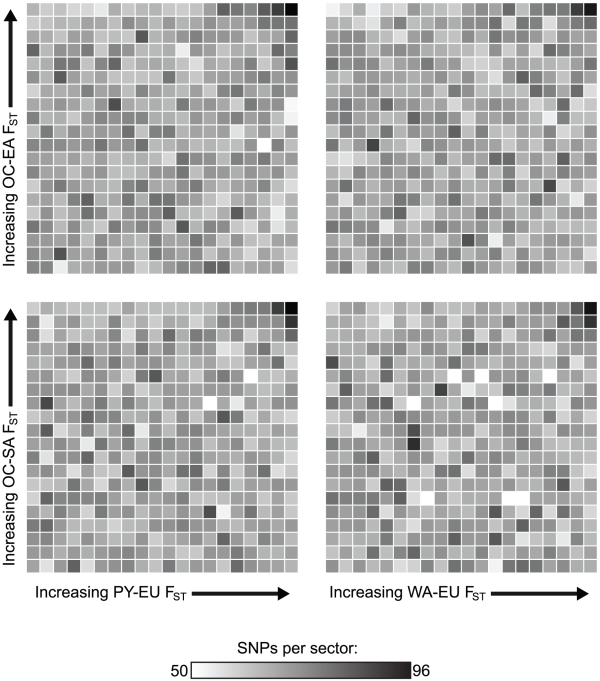
Heat map showing ranks for four divergence comparisons. F_ST_ between Africa (PY or WA) and Europe (EU) is compared with F_ST_ between Oceania (OC) and East Asia (EA) or South America (SA). In each plot, F_ST_ values for a mean of 26,864 randomly selected SNPs have been ranked for both group pairs and are plotted against each other in order of increasing F_ST_; darker shades represent a high proportion of SNPs falling in that particular sector. The sector in the upper right corner of each plot, representing all parallel divergent SNPs at a threshold of 5%, contains more SNPs than any other similarly-sized sector in that plot. These divergence comparisons are phylogenetically independent because the common ancestor of Oceania/Asia/America existed more recently than the common ancestor shared by these three groups and Europe or Africa. Thus, divergent natural selection acting independently both between Europe and Africa and between Oceania and Asia/America is the best explanation for this observation. Convergence due to gene flow would cause a positive correlation throughout each plot, even after excluding high-F_ST_ SNPs, and parallel occurrences of purifying selection would cause the sectors in the lower left corner of each plot to be the darkest; these patterns are not observed.

**Figure 4:**
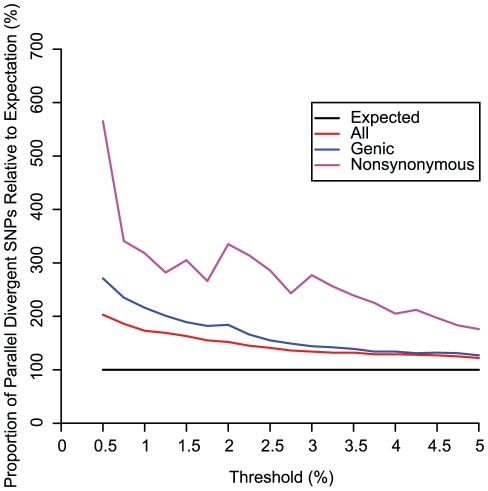
Relative proportions of SNP classes across various thresholds. Relative proportion of parallel divergent SNPs, genic parallel divergent SNPs, and nonsynonymous parallel divergent SNPs are shown for thresholds less than or equal to 5%. All thresholds show an excess of parallel divergent SNPs that is more pronounced for genic SNPs and even more pronounced for nonsynonymous SNPs. As the threshold is lowered, the relative proportion of genic and nonsynonymous SNPs increases faster than for all SNPs combined, suggesting that positive selection is driving the pattern.

In the HGDP dataset, fourteen of the fifteen divergence comparisons showed more parallel divergent SNPs than the expected value of 67.2 (Figure 2). The mean was 82.0 parallel divergent SNPs (range = 58 to 96), a 22% increase over the expected value. Eight divergence comparisons showed a mean of 81.0 or more parallel divergent SNPs, a significant excess according to our simulation results (see below) (Table S1; Figure 2; Figure 3). Four divergence comparisons showed a mean of 91 or more parallel divergent SNPs and therefore were significant even after a conservative Bonferroni correction (Fisher's exact tests, p<0.05). The highest levels of parallel divergence were observed when divergence between Pygmies and Europeans was compared to divergence between Oceanians and South Americans or East Asians. As the threshold for divergence was dropped to more stringent values below 5%, the excess of parallel divergent SNPs increased (Figure 4). There is no comparable excess of parallel conserved SNPs with extremely low divergence in multiple group pairs, suggesting that purifying selection is not the cause of correlations among lineages (Figure 3). The only divergence comparison showing fewer than expected parallel divergent SNPs compares Europe-Oceania F_ST_ to East Asia-South America F_ST_. This is the only divergence comparison that does not include an African population, suggesting that similar selection pressures in African and non-African populations may be driving most of the observed patterns. In addition, as extensive migration between Asia and Oceania could lead to false positives in this divergence comparison, the relative dearth of parallel divergent SNPs suggests that such migration does not have a large effect on our results.

**Figure 5:**
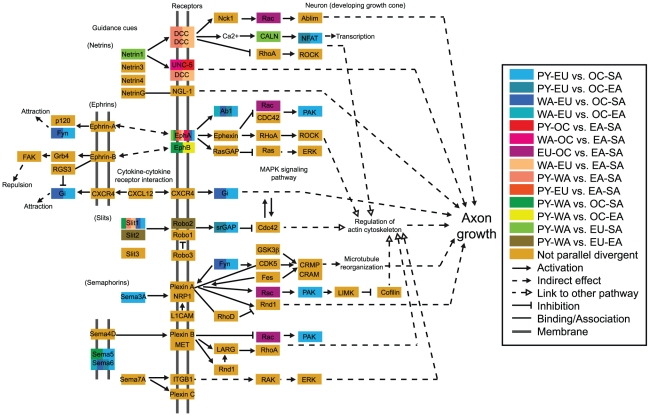
Axon guidance pathway highlighting genes containing parallel divergent SNPs. Most divergence comparisons showed parallel divergent genes in this pathway (enrichment = 1.27), and the PY-EU vs. OC-SA divergence comparison was particularly enriched (enrichment = 2.67) with eleven parallel divergent genes. In this pathway, receptors in the axon growth cone sense guidance cues and signal downstream processes that regulate the cytoskeleton and determine which direction the axon will grow, ultimately influencing the development of neuronal networks. “Positive regulation of axonogenesis” is also an enriched GO term (Table S3). Ephrin receptor *EPHA6* in particular overlaps parallel divergent SNPs in most divergence comparisons and is still observed for “PY-EU vs. EA-SA” and “WA-EU vs. EA-SA” at thresholds as low as 0.5%. Although there are more enriched genes in this pathway than in the pathways in Table S3, the overall per-SNP enrichment is slightly lower for this pathway than for the Table S3 pathways, in part due to differences in mean gene length.

**Figure 6:**
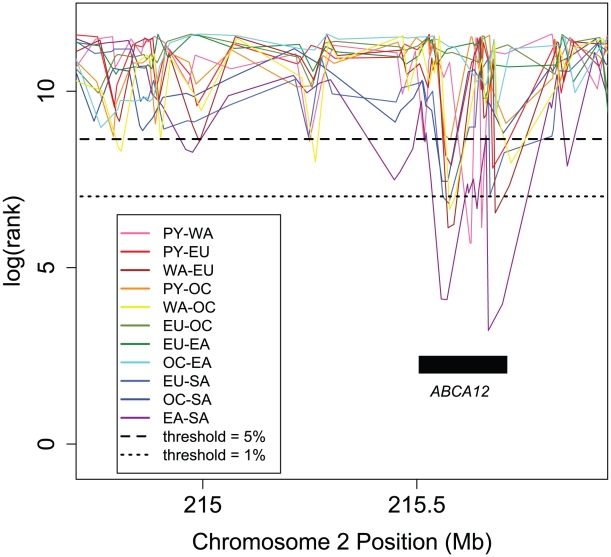
Ranks of F_ST_ in a section of Chromosome 2, illustrating parallel divergence at *ABCA12*. For each group pair, values of F_ST_ at all 111,724 globally polymorphic, intermediate frequency SNPs have been ranked, shown on a log scale (y-axis); here, lower rank corresponds to higher F_ST_. Multiple group pairs show very low ranks due to high F_ST_ across *ABCA12*, with several exceeding a 1% threshold. Mutations in this skin keratinization gene cause harlequin ichthyosis [27]. The orientations of the most extreme divergence comparisons indicate that one allele is common in Africa and South America, while the other is common in Europe, Oceania, and East Asia. Rank lines have been staggered slightly for ease of visualization. ATP-binding cassette (ABC) transporters like this one are enriched among parallel divergent genes (Table S3) and are linked to multi-drug resistance, macular degeneration, breast cancer, diabetes, and other diseases.

Many SNPs fell into narrow regions that were represented in a large number of the 1000 independently generated subsets (Table S2). The mean percentages of both genic and nonsynonymous parallel divergent SNPs were higher than mean for the set of all SNPs in a replicate, a relative excess that increased as the threshold was dropped to more stringent values below 5%, suggesting that positive selection is driving parallel divergence (Figure 4). Genes overlapping parallel divergent SNPs were modestly enriched for diverse functional categories associated with various cell types including neurons, lymphocytes, cancer, and epithelium (Table S1; Table S3; Figure 5). Among the most extreme parallel divergent genes (observed at a threshold of 0.5%) were the skin keratinization gene *ABCA12* [27] (Figure 6); *SH2B1*, which controls serum letpin levels and body weight [28]; *GRM5*, a glutamate receptor associated with schizophrenia [29] and with pigmentation via the closely linked *TYR* [30]; *ATP2A2*, which causes a neuropsychiatric/keratinization disorder [31]; *F13A1*, a coagulation factor linked to numerous cardiovascular diseases and to Alzheimer's [32]; and *IFIH1*, associated with antiviral defense, type 1 diabetes, and psoriasis [33]–[35] (Figure S2). The pleiotropic nature of many of these genes suggests that selection on one trait may have affected the evolution of other traits.

### Orientation Skew among Human Populations

For all divergence comparisons, we calculated the orientation skew, defined as follows. For any divergence comparison, there are two possible allele frequency orientations for a given SNP. In the first pair of groups, one allele is at relatively higher frequency in the first group compared to the second. In the second pair, this allele can either be at relatively higher frequency in the first group or the second, defining the two orientations. The skew is simply the frequency of the more common, or major, orientation for all parallel divergent SNPs, which should be near 0.5 under the neutral expectation that both orientations are equally probable (expected skew is 0.55 for 67 parallel divergent SNPs, following the binomial distribution).

Eleven divergence comparisons showed a significant skew in the mean number of parallel divergent SNPs displaying each orientation across all replicates (major orientation frequency >60%; p<0.05, one-sided comparison to simulation results; Figure 2; Table S1). These results suggest similar selection pressures acting on disparate groups. For example, the most extreme skew (major orientation frequency = 85%) has Europeans diverge from Pygmies in the same direction as East Asians diverge from Oceanians, and there are a myriad of climatological, dietary, and disease variables that could exert similar selective pressures on the two temperate groups relative to the two tropical groups. In fact, four of the five divergence comparisons showing the most parallel divergence show this same general pattern, if Pygmies are considered interchangeable with West Africans and East Asians are considered interchangeable with South Americans (Table S1; Figure 2; Figure 3). This pattern suggests similar selective pressures on Africans and Oceanians relative to Europeans, East Asians, and South Americans. South Americans may carry alleles adapted to temperate climates due to their ancestral migration across Beringia, and they may have lacked adequate time and/or genetic variation to completely re-adapt to a tropical environment. One SNP that fits this hypothesis lies in *DDB1*, which protects the skin from solar UV exposure [36], and is one of the strongest examples of this parallel divergence pattern, with one allele fixed in South America, over 90% in Europe and East Asia, and less than 40% in African and Oceania. Another possible selective pressure driving this pattern may be parasites historically restricted to the Old World tropics; indeed, several immune-related genes like *LRBA* and *LAT* show this parallel divergence pattern even at thresholds well below 5% (Table S2).

### Simulations

To calculate the probability that neutral evolution would generate the observed number of parallel divergent SNPs, we performed nine sets of coalescent simulations, each comprised of 100 full SNP datasets equivalent to our empirical data, under various demographic scenarios including bottlenecks, migration, and growth (Table S4). Some simulations used realistic sets of parameters while others were not realistic for these human populations but tested the effects of diverse demographic events. For each simulated divergence comparison, we calculated the number of parallel divergent SNPs and the orientation skew.

**Figure 7:**
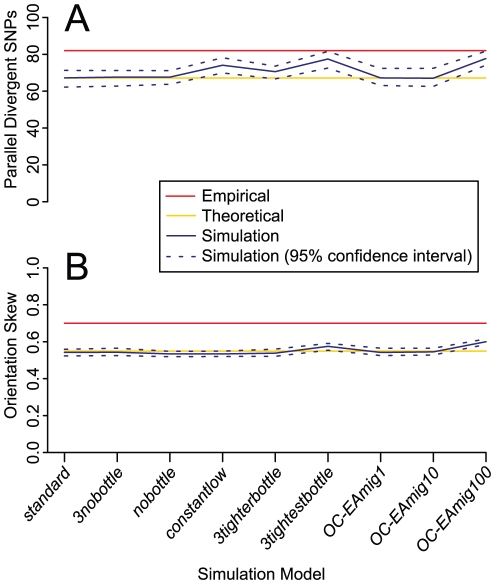
Mean number of parallel divergent SNPs and orientation skew for nine simulation models. Confidence intervals are calculated from 100 independent datasets generated under each model. The empirical mean values are significantly higher than the simulated mean values across all models. A moderate excess of parallel divergent SNPs, although not as high as the empirically observed parallel divergence, was generated by three models: *constantlow*, *3tightestbottle*, and *OC-EAmig100*. These models represent extreme demographic scenarios that are unlikely to accurately approximate the histories of the populations analyzed. Descriptions of the simulated models and their results can be found in Table S4. (A) Mean number of parallel divergent SNPs (B) Mean orientation skew (frequency of major orientation).

Our primary model (*standard*) employed realistic, previously calibrated demographic parameters [37] featuring recent growth in all populations, no migration among populations, and bottlenecks in all populations, including several population sizes under 1000 individuals over 50 generations. Under *standard*, the mean number of parallel divergent SNPs among all simulated divergence comparisons was 67.22 (standard deviation among 1500 divergence comparisons = 7.85; standard deviation among 100 dataset means = 2.37; range of dataset means = 61.27 to 72.28), nearly identical to the theoretical expectation of 67.16 (standard deviation = 7.79; standard deviation of means = 2.01) (Figure 2; Figure 7; Text S1; Figure S3). Out of the 1,500 *standard* simulated divergence comparisons, replicated 10 times each, a mean of 81.0 or more parallel divergent SNPs was observed 2.47% of the time, suggesting that empirical divergence comparisons with at least 81 parallel divergent SNPs exceed the 95% confidence interval. A Bonferroni-corrected significant excess of parallel divergent SNPs (91 or more, theoretically expected 5% of the time if divergence comparisons are independent) was never observed in *standard* (p<0.01). The mean orientation skew was 0.54, and 4.7% of divergence comparisons showed an orientation skew greater than 0.60.

Eight variations on *standard* revealed that our method is in general robust to demographic perturbations, but that extreme demographic events can cause slight excesses of false positives, defined here as values at least 10% greater than the theoretical expectation (Table S4; Figure 7). Eliminating bottlenecks in some or all populations does not cause an excess of false positives, nor does moderate introgression between populations of up to 10 migrants per generation. False positives are produced by extreme bottlenecks of 150 individuals over 50 generations (*3tightestbottle*), extreme migration of 100 migrants per generation (*OC-EAmig100*), or low long-term effective size (*constantlow*). However, no simulation model produced enough false positives to match the empirical results, as mean simulated values for both parallel divergent SNP counts and orientation skew were universally significantly lower than the empirically observed mean results (Figure 7). In the results from two models implemented to represent unrealistically extreme demographic events, *3tightestbottle* and *OC-EAmig100*, a minority of individual simulated divergence comparisons (2 or 3, respectively, out of 15) were comparable to our most significant empirical divergence comparisons (>90 parallel divergent SNPs and/or >0.70 orientation skew) in more than 5% of simulated datasets (Table S4). Presumably, bottlenecks or migration events would have to be even more extreme or prevalent than in these simulations in order to be solely responsible for the mean empirical values across all divergence comparisons. Thus, demographic events are unlikely to be the cause of our empirical results.

## Discussion

The geographic distribution of human genetic diversity shows a pattern of parallel divergence, such that the same variants have undergone exceptionally high divergence repeatedly (Figure 2; Table S2). Both the counts of parallel divergent SNPs and the orientation skews are higher than expected under a neutral framework, which suggests that spatially varying selective pressures are partially responsible. Because observed parallel divergent SNPs did not exceed the expected value at a 5% threshold by more than twofold, the majority of parallel divergent SNPs are probably neutral false positives and not adaptive variants. The relative excess of parallel divergent SNPs increases as the threshold is lowered (Figure 4), so candidates at lower thresholds are more likely to be real adaptive variants, but even at these lower thresholds the evidence for selection on any given SNP reported here (Table S2) is relatively weak in the absence of additional experimental data. Rather than pinpointing specific candidates with high confidence, the main strength of our results is instead to emphasize the broad genomic trend of parallel adaptive divergence.

Our simulations indicate that particular demographic models can cause patterns of parallel divergence similar to those that could be caused by selection. Although this caveat means that our results must be interpreted with caution, demography alone is unlikely to responsible for the empirically observed patterns. None of our models produced mean counts of parallel divergent SNPs or mean orientation skews as high as we observed in the empirical data (Figure 7), and those that did produce unusually high counts of parallel divergent SNPs represented very extreme demographic events (Table S4; Figure 7). Specifically, we only observed an increase in false positives if gene flow occurred on the order of one hundred migrants per generation (*OC-EAmig100*), if bottlenecks occurred with an effective size of 150 individuals over 50 generations in multiple populations (*3tightestbottle*), or if effective size was consistently low with no growth (*constantlow*). None of these three models is particularly realistic for the human populations examined here. While previous analysis suggests that there has been a small amount of admixture between East Asia and Oceania, which could potentially confound three of our divergence comparisons [25], it is unlikely to have been as high as one hundred migrants per generation [38]. Migration between Pygmies and West Africans, or between East Asians and South Americans, would not confound our assumptions of population independence because these populations never appear in different group pairs within the same divergence comparison. Other potential instances of intercontinental gene flow, such as introgression from Europe to South America, do not appear to have contributed to the ancestry of these particular samples, which show negligible evidence of such admixture [25]. Bottlenecks have certainly played an important role in human history, and their magnitude is difficult to estimate with precision, but multiple bottlenecks as extreme as those simulated in *3tightestbottle* would have been unlikely. For example, the Pygmy bottleneck effective size is estimated to have been from ∼500 to several thousand individuals [39], the effective female population size during the colonization of America from Asia has been estimated as ∼1000 individuals [40], and although Oceania may have been founded by very few individuals [38], its cumulative effective size as suggested by modern genetic diversity has been slightly higher than for American populations [25]. As recent human population growth is readily apparent, the constant low effective size simulated by *constantlow* is overly simplistic. Finally, patterns such as an excess of genic and nonsynonymous SNPs (Figure 4) cannot be attributed to demography and imply a prominent contribution by natural selection. Thus, even with the demographic caveats, the highly significant signal of parallel divergence across multiple group pairs suggests that parallel adaptation is an important feature of at least some of these SNPs in some of these populations.

The patterns of parallel adaptive divergence we identified do not reflect classic selective sweeps and therefore suggest more complex modes of selection have shaped human genomic diversity [10]. Our results are consistent with fluctuations in the allele frequencies of standing variation, as in soft sweeps [2], [22], although alleles have rarely actually swept to fixation. The tempered changes at most loci suggest that they encode quantitative polygenic traits that have reached new optima [2], that fitness landscapes fluctuate too rapidly, that the sweeps are still ongoing, and/or that gene flow prevents fixation. Our approach is based on the hypothesis that adaptive variants, or variants closely linked to them, were present in the ancestral human population. Parallel adaptation may also occur via independent adaptive mutations [5], [41]. It is also conceivable that a favorable mutation arising in one population could spread via adaptive introgression to other populations even if gene flow were too low to be detectable at neutral markers. Our method does not preclude the possibility of such newly arisen alleles, although they are less likely to be globally polymorphic. These scenarios still require similar selective pressures acting in multiple distinct populations and thus represent alternate forms of parallel adaptive divergence.

Our method complements existing strategies for detecting intraspecies non-neutral divergence. Most previous studies have focused on identifying loci that differentiate individuals on one continent from all others [4], [6], [8], and thus contribute to the same patterns of population structure generated by neutral processes [2], [7]. While these unique dramatic adaptive events have undoubtedly been important in human evolution, we have shown that phylogenetically orthogonal patterns are also a major component of geographically varying selection. One promising approach for detecting local adaptation is to compare allele frequencies to environmental variables, while controlling for population structure [9]. Our method differs in that we allow for parallel divergence among any group pair regardless of any obvious environmental similarities, and thus we can detect the effects of more cryptic selection pressures. We anticipate that both approaches will be fruitful in uncovering fundamental patterns of local adaptation at the molecular level. Finally, in contrast many other genomic scans for selection [6], a strength of our method is that we do not merely identify the most extreme outliers; rather, we test whether outliers showing parallel divergence are significantly more frequent than expected under neutrality. Future studies on parallel divergence could infer the haplotype backgrounds of selected SNPs via sequencing or denser SNP genotyping, in order to estimate the lengths of the chromosomal regions affected by selection, to further pursue evidence of cryptic gene flow, and to pinpoint causal adaptive SNPs.

In summary, we have demonstrated a statistically significant excess of parallel divergent SNPs in a set of human populations, relative to both the theoretical expectation under neutrality and the values observed in neutral simulations. Although it is difficult to completely rule out the effects of demography on genomic patterns, our simulations and the inferred histories of these populations indicate that a non-adaptive explanation is unlikely. Thus, our results provide statistical support for a major feature of the human evolutionary process: that the same genes are selected independently in multiple environments. Feasible adaptive solutions to selective pressures are therefore limited and are reused in separate lineages [20], [42]. Our approach may gain additional power and lead to new insights with the coming availability of full sequence data from numerous human populations, as well as data from non-human species.

## Methods

We united HGDP populations into six phylogenetically independent groups of at least twenty-five unrelated individuals based on climatic and subsistence designations [9] and population substructure analysis [25]: Pygmy tropical hunter-gather (PY; Biaka and Mbuti), West African tropical horticultural (WA; Mandenka and Yoruba), European temperate agricultural (EU; French, Basque, North Italian, Orcadian, Sardinian, Tuscan), East Asian temperate agricultural (EA; Han, Japanese, Miaozu, Tujia), Oceanian tropical horticultural (OC; Papuan and Melenesian), and South American tropical horticultural (SA; Colombian, Karitiana, Surui). The remaining HGDP populations were excluded either because they showed evidence of admixture between disparate sections of the phylogeny (e.g. Middle Eastern and Central Asian populations), or because they were ecologically or genetically distant from the other populations and the sample size was insufficient for a new group (e.g. San, Pima, Tu). We used an adjusted autosomal SNP dataset for which SNPs with missing data were either abandoned or had missing values estimated based on Hardy-Weinberg equilibrium [43]. We conducted all analyses using only SNPs showing polymorphism in all eleven pairs of analyzed groups (“globally polymorphic”) and showing a global minor allele frequency greater than 0.4 (“intermediate frequency”).

In order to confirm the previously published phylogeny [24], we used the contml package in PHYLIP [44] to determine the evolutionary relationships among our six groups based on allele frequencies at globally polymorphic, intermediate-frequency SNPs. We tested whether the topology with the highest likelihood was significantly better than alternate topologies using Shimodaira and Hasegawa tests [45] with α set at 0.001. Furthermore, for each divergence comparison, we tested whether F_ST_ values were correlated among SNPs that were not divergent; such a correlation would suggest that the pairs were not independent due to gene flow. After ranking each SNP in each divergence comparison according to F_ST_ values, we removed all divergent SNPs (exceeding the 95^th^ F_ST_ percentile) in order to eliminate SNPs under divergent selection. We then regressed the remaining ranks for each pair against each other. We tested whether any divergence comparison showed a significant correlation after a Bonferroni correction (α = 0.05/15 = 0.0033).

For the purpose of calculating linkage disequilibrium in large, panmictic demes, we chose the one population from each group with the largest sample size (Biaka, Mandenka, Sardinian, Karitiana, Papuan, and Han). In each population, we calculated composite pairwise linkage disequilibrium (D) among all globally polymorphic, intermediate frequency SNPs within 10 Mb of each other. We then calculated global D as the average among these populations. For each of 1000 replicates, we randomly selected a combination of globally polymorphic, intermediate frequency SNPs showing pairwise D less than 0.1 (“unlinked”). We did this by randomly selecting unlinked SNPs one by one, each time recalculating the site frequency spectra, defined as the count of SNPs with a minor allele frequency in each of ten equal bins (intervals of 5%), in each group. In order to minimize the population-specific effects of ascertainment and demography, we did not allow the size of any of the ten bins in the site frequency spectrum in any group to deviate from the corresponding bin in another group by more than 5%, unless the difference was under 100; thus, in our final random sample all groups had very similar site frequency spectra (“similar-spectrum”; Figure S1).

For each pair of groups, we calculated pairwise F_ST_ [46] between the two groups at all chosen SNPs and we ranked these F_ST_ values. We defined a SNP to be divergent for a particular group pair if its rank exceeded the designated threshold (top 5% for most analyses). If a SNP was divergent between two phylogenetically independent pairs of groups (a divergence comparison), we considered it to be a parallel divergent SNP. For each divergence comparison, we designated all SNPs as either parallel divergent, divergent in only one pair of groups, or not divergent, and tested the significance of these categories using both Fisher's exact test and comparisons to our simulated results (Text S1). We calculated the mean number per replicate of genic SNPs, nonsynonymous SNPs, and SNPs in genes associated with Gene Ontology (GO) or KEGG pathway terms [47]. We tested whether these categories were enriched for parallel divergent SNPs by using the random sample of globally polymorphic, intermediate-frequency, unlinked, similar-spectrum SNPs as the background, and the parallel divergent SNPs themselves, rather than the genes or regions they overlap, as the test group; this approach should preclude any effect of variation in SNPs per gene or in the probability that a SNP is included for analysis.

We tested for a bias in allele frequency orientation, a binary variable defined by which groups in each divergence comparison have relatively similar allele frequencies at parallel divergent SNPs. In order to examine whether observed orientations strayed from the neutral expectation of equal probability for both orientations, we calculated the mean number of parallel divergent SNPs with each orientation in each divergence comparison, across all replicates. We assessed the deviation from the binomial expectation using both Fisher's exact test and comparisons to our simulated results.

For each of nine demographic models, we used ms [48] to generate 100 independent coalescent-simulated datasets equivalent in size to the empirical HGDP dataset. Each simulation consisted of at least 12,000 unlinked regions representing 100 kb of DNA each, with a per-site mutation rate of 2×10^−9^ and a per-site recombination rate between 0 and 11.25×10^−8^, drawn from a distribution based on the empirical distribution of recombination rates in humans. Our *standard* model was a modified cosi demographic model calibrated using African, European, and Asian HapMap populations [37], splitting the African and Asian populations into two (representing PY and WA), and three (representing EA, OC, and SA) populations, respectively. Additional models were variations on this standard model (Table S4). We excluded all SNPs that were not globally polymorphic and intermediate frequency. For each of ten replicates for each of the 900 independent simulations, we randomly selected 26,864 SNPs to analyze, to match the empirical mean, controlling for LD and site frequency spectra in each random sample as we did with the empirical SNPs. Thus, we analyzed 9000 unique sets of 26,864 simulated SNPs using the same methods we used on the empirical dataset, including the analysis of fifteen distinct divergence comparisons, for a total of 135,000 simulated divergence comparisons.

## Supporting Information

Figure S1 Mean site frequency spectra for all six groups per random sample of independent SNPs. We deliberately constrained the site frequency spectra to be similar in all groups, in order to minimize the effects of ascertainment bias or demography on our results.
(EPS)

Figure S2 Ranks of F_ST_ in a section of Chromosome 2, illustrating parallel divergence at *IFIH1*. For each group pair, values of F_ST_ at all 111,724 globally polymorphic, intermediate frequency SNPs have been ranked, shown on a log scale (y-axis). The phylogenetically independent group pairs PY-EU and EA-SA both show very low ranks due to high F_ST_ at two SNPs in *IFIH1*. Polymorphisms in this gene are associated with antiviral defense, type 1 diabetes, and psoriasis [33]–[35]. The EU-SA divergence shows the lowest rank, as the orientation is: (EU/EA, PY/SA).
(EPS)

Figure S3 Heat map showing ranks for four simulated divergence comparisons. F_ST_ between Africa (PY or WA) and Europe (EU) is compared with F_ST_ between Oceania (OC) and East Asia (EA) or South America (SA). This figure is equivalent to Figure 3 except that it uses neutral simulated data from the *standard* model (Table S4) instead of empirical data, and the scale of shades is not identical; see Figure 3 for further explanation. The sector in the upper right corner of each plot represents all parallel divergent SNPs at a threshold of 5%. Unlike the empirical data (Figure 3), this sector does not show an excess of SNPs in any plot, suggesting that neutral demographic processes will not cause false positives in our analysis.
(EPS)

Table S1 Parallel divergence in all fifteen divergence comparisons.
(PDF)

Table S2 All sites showing parallel divergence in at least one replicate.
(XLS)

Table S3 Enriched Gene Ontology Biological Process (GO BP) terms and KEGG pathways among parallel divergent SNPs.
(PDF)

Table S4 Results from nine coalescent simulation models representing various demographic scenarios.
(PDF)

Text S1 Formal contingency table methodology for detecting parallel adaptive divergence.
(PDF)
