# Supplementary material for: Parallel Adaptive Divergence among Geographically Diverse Human Populations
Source: PLoS Genet. 2011 Jun 16;7(6):e1002127. doi: 10.1371/journal.pgen.1002127 (PMC3116918; doi:10.1371/journal.pgen.1002127)
Supplement: Table S1 — Parallel divergence in all fifteen divergence comparisons. (PDF) [file pgen.1002127.s004.pdf]

Table S1
Parallel divergence in all fifteen divergence comparisons.

**Divergence Comparison**[a]	**Parallel Divergent SNPs**[b]	**Major Orientation**[c]	**Skew**[d]	**GO BP term**[e]
PY-EU vs. OC-SA	96.1**	PY/OC, EU/SA	0.61*	n/a
PY-EU vs. OC-EA	93.8**	PY/OC, EU/EA	0.85**	n/a
PY-OC vs. EA-SA	92.8**	PY/SA, OC/EA	0.83**	homeostatic process; cell cycle
WA-EU vs. OC-SA	92.4**	WA/OC, EU/SA	0.62*	synaptic transmission
WA-EU vs. OC-EA	89.5*	WA/OC, EU/EA	0.82**	proteolysis
PY-WA vs. EU-SA	86.6*	PY/SA, WA/EU	0.71*	n/a
WA-OC vs. EA-SA	86.1*	WA/SA, OC/EA	0.83**	n/a
PY-WA vs. EU-EA	85.6*	PY/EA, WA/EU	0.60	cognition; sensory perception
PY-EU vs. EA-SA	79.1	PY/SA, EU/EA	0.82**	transmembrane receptor protein tyrosine kinase signaling pathway; protein phosphorylation
PY-WA vs. OC-EA	75.8	PY/OC, WA/EA	0.59	n/a
PY-WA vs. EU-OC	75.8	PY/OC, WA/EU	0.68*	n/a
PY-WA vs. OC-SA	75.1	PY/SA, WA/OC	0.55	regulation of transcription, DNA-dependent; proteolysis
PY-WA vs. EA-SA	73.9	PY/SA, WA/EA	0.64*	neuron projection development
WA-EU vs. EA-SA	69.1	WA/SA, EU/EA	0.78*	cell surface receptor linked signal transduction; transmembrane receptor protein tyrosine kinase signaling pathway; protein phosphorylation; homeostatic process
EU-OC vs. EA-SA	58.1	EU/SA, OC/EA	0.57	n/a

[a]F_{ST} at all SNPs between the first pair of groups is compared to F_{ST} at all SNPs between the second pair of groups
[b]Mean number of parallel divergent SNPs in 1000 replicates (expected number is 67.2).
[c]More frequently observed of the two possible orientations. Each orientation unites each group from the first divergence comparison with one of the groups from the second divergence comparison, according to which groups had relatively similar allele frequencies.
[d]The proportion of parallel divergent SNPs showing the major orientation.
[e]Enriched Gene Ontology Biological Process terms. All terms showing at least 2-fold enrichment and a mean of at least three SNPs per replicate for that divergence comparison are shown, with redundant terms eliminated. “n/a” indicates no terms met the criteria.
*p<0.05 in comparison to simulation *standard*
**p<0.05 in comparison to simulation *standard* and in Bonferroni-corrected Fisher’s exact test
