# Supplementary material for: Parallel Adaptive Divergence among Geographically Diverse Human Populations
Source: PLoS Genet. 2011 Jun 16;7(6):e1002127. doi: 10.1371/journal.pgen.1002127 (PMC3116918; doi:10.1371/journal.pgen.1002127)
Supplement: Table S3 — Enriched Gene Ontology Biological Process (GO BP) terms and KEGG pathways among parallel divergent SNPs. (PDF) [file pgen.1002127.s006.pdf]

Table S3

Enriched Gene Ontology Biological Process (GO BP) terms and KEGG pathways among parallel divergent SNPs.

Category[a]	Type	Mean PD SNPs[b]	Total PD Genes[c]	Enrichment[d]
Protein folding	GO BP	3.6	9	5.06
Protein heterooligomerization	GO BP	3.5	9	4.31
Integrin-mediated signaling pathway	GO BP	4.9	15	2.55
Cholesterol metabolic process	GO BP	3.3	17	2.44
Positive regulation of axonogenesis	GO BP	3.4	8	2.22
DNA repair	GO BP	4.2	14	2.21
Protein oligomerization	GO BP	6.3	19	2.19
Epithelial cell differentiation	GO BP	3.3	8	2.05
ABC transporters	KEGG	3.7	6	3.16
Chronic myeloid leukemia	KEGG	3.3	7	2.79
B cell receptor signaling pathway	KEGG	3.2	8	2.33
Prostate cancer	KEGG	3.6	9	2.28
Ubiquitin mediated proteolysis	KEGG	3.7	13	2.25
Olfactory transduction	KEGG	3.3	7	2.21
Neurotrophin signaling pathway	KEGG	4.3	11	2.14
Gap junction	KEGG	6.6	12	2.08
T cell receptor signaling pathway	KEGG	3.8	13	2.05

[a]All categories showing at least 2-fold enrichment and a mean of at least three SNPs per replicate are shown, with redundant terms eliminated. While not significant when corrected for multiple tests, these categories provide an overview of the characteristics of parallel divergent genes. Some categories (e.g. “cholesterol metabolic process”, “B cell receptor signaling pathway”) are consistent with obvious geographically varying selective pressures such as diet and disease, while others are less intuitive.

[b]Mean number of parallel divergent SNPs per replicate that overlap a gene in this category, among all divergence comparisons.

[c]Total number of genes in this category that contain a parallel divergent SNP in at least one replicate.

[d]Ratio of the mean proportion of parallel divergent SNPs in a replicate that overlap a gene in this category to the proportion of all independent SNPs in a replicate that that overlap a gene in this category.
