# Supplementary material for: Parallel Adaptive Divergence among Geographically Diverse Human Populations
Source: PLoS Genet. 2011 Jun 16;7(6):e1002127. doi: 10.1371/journal.pgen.1002127 (PMC3116918; doi:10.1371/journal.pgen.1002127)
Supplement: Table S4 — Results from nine coalescent simulation models representing various demographic scenarios. (PDF) [file pgen.1002127.s007.pdf]

Table S4

Results form nine coalescent simulation models representing various demographic scenarios.

Model	Description[a]	Mean Parallel Divergent SNPs (95% CI)[b]	Mean Skew (95% CI)[c]	Divergence Comparison with Highest Parallel Divergence (DCHPD)[d]	Parallel Divergent SNPs of DCHPD (95% CI)[e]	Most Skewed Divergence Comparison (MSDC)[f]	Skew of MSDC (95% CI)[g]
standard	cosi-based parameters. WA and PY follow Africa; EU follows Europe; EA, OC, SA follow Asia. Bottlenecks but N > 500 at all times	67.2 (62.2,71.3)	0.54 (0.52,0.56)	PY-WA vs. EU-EA	68.6 (56.7,82.7)	PY-WA vs. OC-EA	0.55 (0.51,0.64)
3nobottle	*standard* but no bottlenecks for out-of-Africa or for PY, OC, or SA	67.6 (62.8,71.2)	0.54 (0.53,0.57)	WA-OC vs. EA-SA	68.5 (56.8,79.8)	PY-WA vs. EU-SA	0.55 (0.50,0.62)
OC-EAmig1	*standard* but gene flow from EA to OC, 4Nm = 1	67.2 (63.1,72.3)	0.54 (0.53,0.57)	PY-WA vs. EU-OC	69.0 (56.1,82.9)	PY-OC vs. EA-SA	0.55 (0.50,0.65)
OC-EAmig10	*standard* but gene flow from EA to OC, 4Nm = 10	67.0 (62.6,72.4)	0.55 (0.53,0.57)	PY-WA vs. EU-EA	69.1 (55.7,80.3)	WA-OC vs. EA-SA	0.56 (0.50,0.63)
OC-EAmig100	*standard* but gene flow from EA to OC, 4Nm = 100	77.7 (74.1,81.7)	0.60 (0.59,0.62)	EU-OC vs. EA-SA	91.7 (77.3,104.3)	EU-OC vs. EA-SA	0.83 (0.77,0.90)
constantlow	*standard* but no bottlenecks or population growth, constant N = 10,000	74.1 (69.9,78.1)	0.53 (0.52,0.55)	PY-WA vs. OC-SA	78.0 (66.1,88.2)	EU-OC vs. EA-SA	0.55 (0.50,0.64)
nobottle	*standard* but no bottlenecks	67.6 (63.8,71.2)	0.53 (0.52,0.55)	PY-EU vs. SA-EA	68.4 (58.6,79.4)	PY-EU vs. SA-OC	0.54 (0.50,0.60)
3tighterbottle	*standard* but tighter bottlenecks for PY, OC, and SA (N = 350 for 50 generations)	70.6 (66.7,73.6)	0.54 (0.52,0.56)	PY-EU vs. OC-SA	72.9 (64.4,84.3)	PY-EU vs. EA-SA	0.55 (0.50,0.63)
3tightestbottle	*standard* but even tighter bottlenecks for PY, OC, and SA (N = 150 for 50 generations)	77.5 (72.5,81.5)	0.57 (0.55,0.59)	PY-EU vs. OC-SA	90.4 (79.3,101.5)	PY-EU vs. EA-SA	0.64 (0.58,0.72)

[a]Brief description of each model. All models join populations in the same order, SA-EA-OC-EU-WA-PY, at the same time points. Parameters follow the cosi model calibrated with HapMap data unless otherwise indicated. All bottlenecks last for 50 generations.

[b]Mean number of parallel divergent SNPs across all divergence comparisons, with 95% confidence intervals.

[c]Mean proportion of parallel divergent SNPs showing the major orientation across all divergence comparisons, with 95% confidence intervals.

[d]Divergence comparison (out of 15) showing the highest number of parallel divergent SNPs

[e]Mean number of parallel divergent SNPs for the divergence comparison identified in the previous column, with 95% confidence intervals.

[f]Divergence comparison (out of 15) showing the highest major orientation frequency.

[g]The proportion of parallel divergent SNPs showing the major orientation for the divergence comparison identified in the previous column, with 95% confidence intervals.
