# Supplementary material for: Parallel Adaptive Divergence among Geographically Diverse Human Populations
Source: PLoS Genet. 2011 Jun 16;7(6):e1002127. doi: 10.1371/journal.pgen.1002127 (PMC3116918; doi:10.1371/journal.pgen.1002127)
Supplement: Text S1 — Formal contingency table methodology for detecting parallel adaptive divergence. (PDF) [file pgen.1002127.s008.pdf]

Formal contingency table methodology for detecting parallel adaptive divergence.

SNPs in a divergence comparison between group pairs A and B at threshold θ can be represented in a 2x2 table:

$$\begin{array}{|cc|c} \hline n_{11} & n_{12} & A_1 \\ n_{21} & n_{22} & A_2 \\ \hline B_1 & B_2 & T \end{array}$$

Where:
n_{11} = count of parallel divergent SNPs
n_{12} = count of SNPs divergent in group pair A but not group pair B
n_{21} = count of SNPs divergent in group pair B but not group pair A
n_{22} = count of SNPs not divergent in either group pair
A_1 = count of SNPs divergent in group pair $A = \theta T = n_{11} + n_{12}$
A_2 = count of SNPs not divergent in group pair $A = (1 - \theta)T = n_{21} + n_{22}$
B_1 = count of SNPs divergent in group pair $B = \theta T = n_{11} + n_{21}$
B_2 = count of SNPs not divergent in group pair $B = (1 - \theta)T = n_{12} + n_{22}$
T = Total count of SNPs = $A_1 + A_2 = B_1 + B_2$

The neutral expectation for n_{11} (based on the Mantel-Haenszel Test) is:

$$Mean\{n_{11}\} = \frac{A_1 B_1}{T} = \theta^2 T$$

$$Variance\{n_{11}\} = \frac{A_1 A_2 B_1 B_2}{TT(T-1)} = \frac{(\theta(1-\theta)T)^2}{T-1}$$

Parallel adaptive divergence is inferred if n_{11} if significantly higher than expected under neutrality. In this study, we used both Fisher's exact test and comparisons to our simulated data; other tests for tables of counts (e.g. χ^2) would also be appropriate.

The standard deviation is the square root of the variance. The standard deviation for the mean among a set of divergence comparisons can be approximated by the standard error (i.e. dividing by the square root of the number of divergence comparisons), although this approach should be used with caution because divergence comparisons are not necessarily independent of each other.
